# Effect of Heavy-Metal-Resistant PGPR Inoculants on Growth, Rhizosphere Microbiome and Remediation Potential of *Miscanthus × giganteus* in Zinc-Contaminated Soil

**DOI:** 10.3390/microorganisms11061516

**Published:** 2023-06-07

**Authors:** Anna Muratova, Sergey Golubev, Valeria Romanova, Irina Sungurtseva, Asil Nurzhanova

**Affiliations:** 1 Institute of Biochemistry and Physiology of Plants and Microorganisms, Saratov Scientific Centre of the Russian Academy of Sciences (IBPPM RAS), 410049 Saratov, Russia; sngolubev@rambler.ru (S.G.); airinmind@yandex.ru (I.S.); 2 Institute of Fundamental Medicine and Biology, Kazan (Volga Region) Federal University, 420021 Kazan, Russia; avonamora-94@mail.ru; 3 Institute of Plant Biology and Biotechnology, Almaty 050040, Kazakhstan; gen_asil@mail.ru

**Keywords:** *Miscanthus × giganteus*, *Chitinophaga* sp., *Mycolicibacterium* sp., PGPR, zinc, rhizosphere microbial communities, bacterial diversity, phytoremediation

## Abstract

Microbial-assisted phytoremediation is considered a more effective approach to soil rehabilitation than the sole use of plants. *Mycolicibacterium* sp. Pb113 and *Chitinophaga* sp. Zn19, heavy-metal-resistant PGPR strains originally isolated from the rhizosphere of *Miscanthus × giganteus*, were used as inoculants of the host plant grown in control and zinc-contaminated (1650 mg/kg) soil in a 4-month pot experiment. The diversity and taxonomic structure of the rhizosphere microbiomes, assessed with metagenomic analysis of rhizosphere samples for the 16S rRNA gene, were studied. Principal coordinate analysis showed differences in the formation of the microbiomes, which was affected by zinc rather than by the inoculants. Bacterial taxa affected by zinc and the inoculants, and the taxa potentially involved in the promotion of plant growth as well as in assisted phytoremediation, were identified. Both inoculants promoted miscanthus growth, but only *Chitinophaga* sp. Zn19 contributed to significant Zn accumulation in the aboveground part of the plant. In this study, the positive effect of miscanthus inoculation with *Mycolicibacterium* spp. and *Chitinophaga* spp. was demonstrated for the first time. On the basis of our data, the bacterial strains studied may be recommended to improve the efficiency of *M. × giganteus* phytoremediation of zinc-contaminated soil.

## 1. Introduction

The interest in nonfood perennial plant species that can be used as alternative energy sources (biofuels) continues unabated. *Miscanthus × giganteus* Greef et Deu. (Poaceae family), known as the giant miscanthus, a sterile hybrid of *Miscanthus sinensis* and *Miscanthus sacchariflorus*, is a promising bioenergetic species owing to its high biomass yield and low production costs [1,2,3]. Planting this crop on contaminated soils unsuitable for food production, in addition to yielding biomass for biofuel production, can simultaneously solve the soil remediation problem [3,4,5]. *M. × giganteus* is resistant to various levels of heavy metal pollution of soil and can be applied for soil cleanup [6,7]. At the same time, the microorganisms associated with its root zone can affect both biomass production and soil cleanup, promoting plant growth directly (through improved plant nutrition and phytohormone production) and/or indirectly (through a change in the bioavailability of metals) [8,9]. On the basis of this, the microbe-assisted phytoremediation is considered a more preferable approach than the sole use of plants [10,11,12].

The accumulation of heavy metals by plants depends on many factors, including soil pH, the degree of metal oxidation, and the species of associative bacteria. Plant growth-promoting rhizobacteria (PGPR), through their abilities to enhance plant growth and affect pollutant solubility and bioavailability, can play an important role in the phytoremediation of soils contaminated with heavy metals [9,13,14]. PGPR traits such as nitrogen fixation, phosphorous mobilization, and siderophore production are responsible for improving plant nutrition, phytohormone production—for regulation of plant growth and development, capacities of inducing plant defense mechanisms—for protecting hosts from exposure to biotic and abiotic stress factors [8,9]. PGPR can increase the bioavailability of heavy metals by promoting their uptake by the plant. Examples of improving plant growth and enhancing the phytoremediation of heavy-metal-contaminated soil are summarized in a review [10]. However, the PGPR applications not always increase the uptake of metals by plants and soil cleanup. Bacteria affect metal phytoaccumulation by releasing extracellular organic acids and immobilizers of a polymeric nature (lipids, nucleic acids, proteins, polysaccharides, and biosurfactants) [9,15]. These substances play a significant role in the formation of complexes with metals, thereby reducing their bioavailability [9,11]. The main mechanisms for reducing metal mobility in PGPR are bioaccumulation and biosorption [9,16]. The first is carried out both through active and passive transport of metals into the bacterial cell; the second, through such processes as precipitation, sequestration, and transformation. Examples of reduced metal phytoaccumulation under the influence of heavy-metal-resistant PGPR strains have been summarized [17].

There are several research reports devoted to the evaluation of rhizosphere microbial communities of *Miscanthus* spp. [18,19,20,21,22,23,24,25]. Researchers are also interested in the prospect of using miscanthus inoculation with PGPR to promote plant growth [26,27,28,29,30,31]. However, this area of research is still far from saturated with information. The selection of promising bacterial strains from the root zone of the target plant species, followed by testing of their efficiency as biofertilizing inoculants, remains a relevant approach to identifying key points of plant–microbe interactions under specific habitat conditions, which is a necessary step in microbial biopreparation development.

The objective of this study was to examine two bacterial strains, previously isolated from the heavy-metal-contaminated rhizosphere of *Miscanthus × giganteus* [32], to evaluate their effect on the growth, rhizosphere microbiome, and phytoremediation potential of the host plant growing in zinc-contaminated soil.

## 2. Materials and Methods

### 2.1. Soil Preparation

Kastanozem soil (WRB: Haplic and Gypsic Kastanozems), typical of the arid area of the Saratov Trans-Volga region (near the town of Engels), was used in this study. Before vegetation experiments, the soil was characterized for the following variables: particle size distribution, moisture capacity, pH, mineral nitrogen content, and phosphorus content. According to the analyses: the soil contained 14.4 and 10.7 mg/kg of ammonium and nitrate nitrogen, respectively, and 253.3 mg/kg P_2_O_5_; total moisture capacity was 39%; the pH was 7.2; and the fraction of particles: >3 mm, 0.026%; 1–3 mm, 0.036%; 0.5–1 mm, 0.169%; 0.25–0.5 mm, 4.229%; ˂0.25 mm, 95.450%.

Before the pot experiment, air-dried soil was sieved through a 7 mm mesh net and was treated with a zinc solution (ZnSO_4_·7H_2_O, >99% purity, Reakhim, Moscow, Russia) in deionized water to a final Zn^2+^ ion concentration of 1650 mg/kg, corresponding to a level 15-fold higher than the approximate permissible level (APC_Zn_ = 110 mg/kg [33]). The control soil was treated with an equal volume of distilled water. The treated contaminated and uncontaminated soil (humidity 45%) was kept for 3 days to achieve even distribution of the pollutant; then, soil was sampled for initial analyses and packaged (1 kg) in 2-L plastic pots.

### 2.2. Rhizobacterial Strains

In the course of previous studies, from the heavy-metal-contaminated rhizosphere of *M. × giganteus* 76 bacterial strains have been isolated [32,34]. The bacterial strains, marked as Zn19 and Pb113, were isolated from the heavy-metal-contaminated rhizosphere of *M. × giganteus* by using the Luria-Bertani (LB) agar medium containing the water-soluble zinc salt (ZnSO_4_·7H_2_O) or lead salt (Pb(NO_3_)_2_) at a final metal ion concentration of 0.5 mmol/L. The isolates were tested for metal resistance; to this end, they were grown on the LB agar medium containing Zn^2+^ or Pb^2+^ (0.5–5 mmol/L). The strains were tested for their ability to fix atmospheric nitrogen, produce the phytohormone indole-3-acetic acid (IAA) [7] and siderophores [35,36], and dissolve phosphates [37]. After the abilities had been confirmed, the strains were subjected to identification. The primary taxonomic identification of strains Pb113 and Zn19 was carried out on the basis of 16S rRNA gene sequence analysis using the EzBioCloud server [38]. For details, see [39]. The Pb113 and Zn19 16S rRNA gene sequences are deposited in the GenBank under accession numbers OQ680140 and OQ680520, respectively. The strains are deposited in the IBPPM RAS Collection of Rhizosphere Microorganisms (https://collection.ibppm.ru, accessed on 30 May 2023).

### 2.3. Experimental Design

One-year-old rhizomes of *Miscanthus × giganteus* J.M. Greef, Deuter ex Hook, Renvoize were obtained from the Institute of Plant Biology and Biotechnology in Almaty, Kazakhstan, and were used in the research. For planting, experimental pots were filled by pouring expanded clay (300 mL per pot; granule diameter 1.5–2.0 cm) as a drainage on the bottom of the vessel. The drainage was closed with gauze, and river sand (300 g per pot) was poured on top and covered again with gauze. Then, the pots were filled with soil (1000 g dry weight per pot). Miscanthus rhizomes were planted in the prepared pots (2 pieces per vessel). After planting, the soil surface was covered with sand (100 g per pot). All experiments were performed in triplicate. The pots with uncontaminated soil (without Zn treatment) were used as controls. Soil moisture in the pots was maintained at 50% of the full moisture capacity by daily watering with standing tap water, the need for which was determined by weighing the pots. The plants were grown in a greenhouse at 24–28/20–22 °C and natural illumination for 4 months (from May to September 2021).

Inoculation of one-week-old seedlings with heavy-metal-resistant PGPR strains Zn19 and Pb113 was carried out as follows. Two-day-old microbial biomass was collected from R2A agar medium [40], washed with saline twice, and resuspended in the plant watering liquid. Inoculation of plants was carried out by watering of the corresponding variants with a microbial suspension to the final concentration of cells in the soil of at least 10^7^ cells per gram. The plants were inoculated once.

### 2.4. Plant Biomass Measurement

To control the changes in plant biomass, the weight of the rhizomes planted was measured before planting. In the course of the experiment, the plant tillering and height were measured monthly. At the end of cultivation, the plants were removed from the pots, and the shoots and roots were separated from the rhizomes. Samples of young roots with the remaining attached rhizospheric soil were taken for microbiological analysis. The remaining roots were separated from the rhizomes, washed free of soil with tap water, weighed and dried to constant weight in an oven at 70 °C. The remaining rhizomes were also washed free of soil with tap water and weighed.

### 2.5. Measurement of Metal Content in Plant Biomass

The content of Zn in plant biomass was determined by atomic absorption spectroscopy as reported previously [41].

The translocation factor (TF) was calculated for each treatment. The TF, reflecting the plant’s ability to transport and accumulate metals into aboveground biomass, was calculated as the ratio of the metal concentration in the shoots to the metal concentration in the roots.

### 2.6. 16S rRNA Gene-Based Metagenomic Analyses of Rhizosphere Soil and Bioinformatics

Extraction and purification of soil DNA, 16S rRNA sequencing library preparation, 16S rRNA amplicon sequencing, quality filtering of reads, OTU taxonomy, characterization of the richness and evenness of bacterial communities, assessment of the similarity between the microbial composition of samples were carried out as described in [42]. Raw reads are deposited in the NCBI Sequence Read Archive (SRA) under BioProject accession number PRJNA973256.

### 2.7. Statistics

All the experimental data obtained were subjected to statistical processing, calculating the average values, for comparison of which the standard deviation and the confidence interval at *p* ≤ 0.05 were used. Calculations were performed in Microsoft Excel 2016 (Microsoft, Redmond, WA, USA). To compare the average values, identify the effect of soil pollution and/or microbial inoculation factors, analysis of variance and Spearman’s rank correlations (*r_s_*) were used, which were performed in Statistica 13.3.721.1 (TIBCO Software Inc. 2017, Statsoft, Moscow, Russia).

## 3. Results

### 3.1. Characteristics of Bacterial Inoculants

Previously isolated strains Pb113 and Zn19 were studied for their resistance to Pb^2+^ and Zn^2+^ and for plant growth promoting potential, manifested through nitrogen fixation, phosphorous mobilization, and synthesis of siderophores and phytohormones. These isolates manifested combined properties of PGPR and resistance to metals, were selected (Table 1).

To identify Pb113 and Zn19, their 16S rRNA gene sequences were determined: 1442 and 1449 bp, respectively.

**Figure 1:**
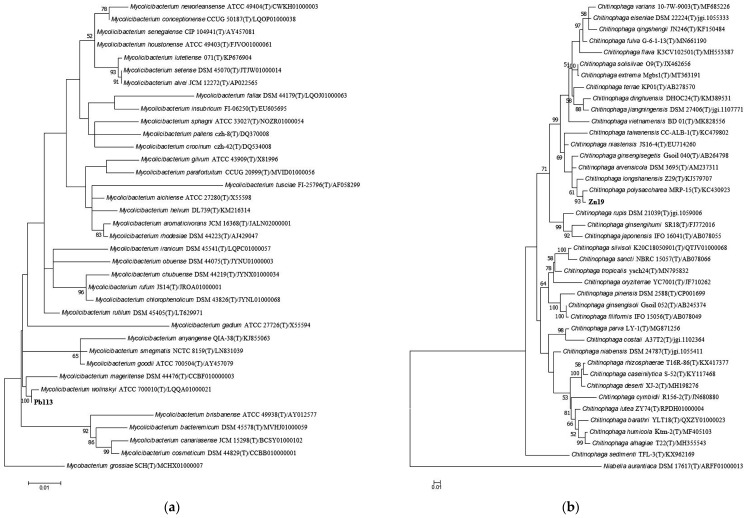
Maximum-likelihood phylogenetic trees based on the 16S rRNA gene sequences from the strains under study—Pb113 (**a**) and Zn19 (**b**)—and their phylogenetic neighbors. The search for the best evolutionary model and the reconstruction of phylogenetic trees were conducted in MEGA7 [43]. Nonuniformity of evolutionary rates among sites was imitated by using a discrete Gamma distribution (+G) and by assuming that a certain fraction of sites was evolutionarily invariable (+I). Bootstrap values (≥50%) based on 1000 replications are shown at branch nodes. GenBank numbers of sequences are given after strain names. Bar, 0.01 substitutions per nucleotide position. (**a**) Tamura–Nei model [44] + G (gamma shape parameter = 0.1000) + I (87.47%); (**b**) Kimura 2-parameter model [45] + G (gamma shape parameter = 0.1281) + I (66.42%).

The homolog search for the 16S rRNA gene sequence from Pb113 (Figure 1a) showed that this strain is closely related to members of the *Mycolicibacterium* genus, forming a separate clade with the type strain of *M. wolinskyi*. The pairwise sequence similarity of Pb113 with *M. wolinskyi* ATCC 700010T was 99.86%. Yet, seven more type strains had pairwise sequence similarities with the microorganism under study above 98.65%, recommended as the species boundary cutoff [35]: *M. mageritense* DSM 44476^T^ (99.38%), *M. rutilum* DSM 45405^T^ (99.24%), *M. chlorophenolicum* DSM 43826^T^ (98.82%), *M. rufum* JS14^T^ (98.82%), *M. rhodesiae* DSM 44223^T^ (98.75%), *M. iranicum* DSM 45541^T^ (98.74%), and *M. goodii* ATCC 700504^T^ (98.68%).

As shown by the phylogenetic analysis, Zn19 occupies a robust position among the bacteria of the genus *Chitinophaga* and is most closely related to *C. polysaccharea* (Figure 1b). The pairwise sequence similarity of Zn19 to *C. polysaccharea* MRP-15^T^ was 99.00%, whereas with other relatives, it was below the threshold for species demarcation. Thus, it can be concluded that Pb113 belongs to the *Mycolicibacterium* genus and that Zn19 belongs to the *Chitinophaga* genus.

### 3.2. Effect of Bacterial Inoculants and Zn on the Growth Performances of M. × giganteus

**Figure 2:**
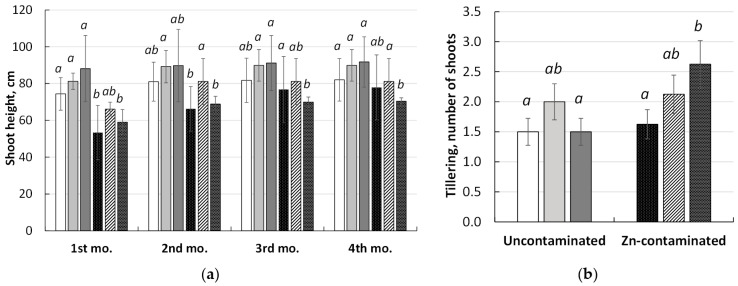
Average height (**a**) and tillering (**b**) of inoculated and non-inoculated *M. × giganteus* grown in uncontaminated and Zn-contaminated soil. 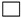
—uncontaminated, non-inoculated; 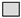
—uncontaminated + *Mycolicibacterium* sp. Pb113; 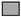
—uncontaminated + *Chitinophaga* sp. Zn19; 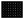
—Zn-contaminated, non-inoculated; 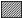
—Zn-contaminated + *Mycolicibacterium* sp. Pb-113; 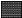
—Zn-contaminated + *Chitinophaga* sp. Zn19. Values represent means, and bars represent confidence interval (n ≥ 3); different letters mean significant difference between treatments at *p* ≤ 0.05.

The Zn contamination of soil and inoculation affected *M. × giganteus* height and tillering (Figure 2).

Owing to the large variation in the height of shoots of different ages, there were no significant differences between treatments. However, a clear trend of zinc inhibition of the shoot height was observed (Figure 2a). The inoculation effect on the shoot height depended on the microorganism used and on the soil treatment. Thus, in uncontaminated soil, there was a trend to increase the shoot height under the influence of *Chitinophaga* sp. Zn19 and *Mycolicibacterium* sp. Pb113, and in Zn-contaminated soil, that was only under the influence of *Mycolicibacterium* sp. Pb 113. Plant tillering was promoted significantly (62%) by *Chitinophaga* sp. Zn19 only (Figure 2b).

**Figure 3:**
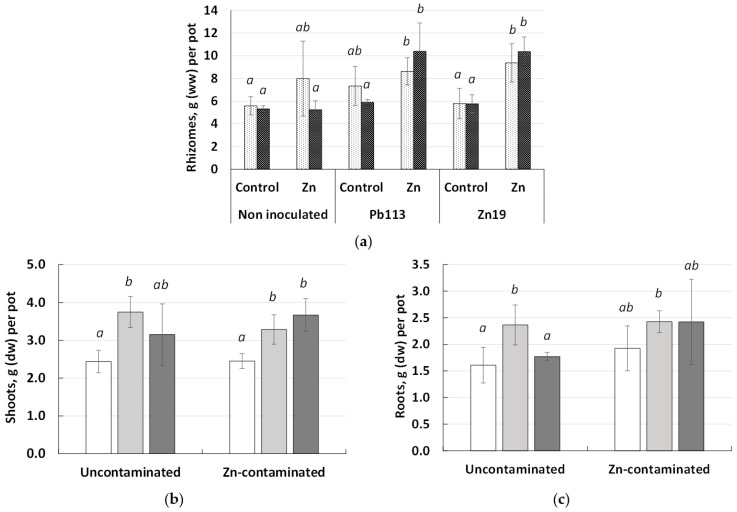
Biomass of rhizomes (**a**), shoots (**b**), and roots (**c**) of inoculated and non-inoculated *M. × giganteus* grown in uncontaminated and Zn-contaminated soil. 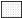
—rhizome biomass at planting; 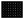
—rhizome biomass at the end of cultivation; 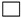
—non-inoculated control; 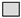
—*Mycolicibacterium* sp. Pb113; 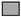
—*Chitinophaga* sp. Zn19. Values represent means, and bars represent confidence interval (n ≥ 3); different letters mean significant difference between treatments at *p* ≤ 0.05.

The changes in miscanthus biomass under the influence of the rhizobacterial inoculants and zinc are illustrated in Figure 3.

Despite the attempts to evenly distribute the biomass of planting material (rhizomes) at the beginning of the experiment, there still was some differences between the pots. Therefore, when the final biomass of the rhizomes was measured, the initial planting weight of the rhizomes was also taken into account (Figure 3a). According to the data obtained, no significant change in wet rhizome weight was found in the uncontaminated control soil. There was only a tendency to decrease biomass under the influence of *Mycobacterium* sp. Pb 113. In Zn-contaminated soil, there was no significant change in wet rhizome weight, but there was a tendency to decrease the biomass of the rhizomes of the non-inoculated plants.

Plant inoculation with *Mycolicibacterium* sp. Pb113 caused a significant increase in the shoot (Figure 3b) and root (Figure 3c) biomass of miscanthus in uncontaminated soil by 54 and 47%, and in Zn-contaminated soil, by 34 and 26%, respectively. Applying *Chitinophaga* sp. Zn19 did not cause a significant change in plant biomass (Figure 3b,c) in uncontaminated soil but increased shoot biomass accumulation by 50% in Zn-contaminated soil (Figure 3b).

### 3.3. Effect of Bacterial Inoculants and Zn on Rhizosphere Microbial Communities of M. × giganteus

#### 3.3.1. Diversity of Rhizosphere Microbial Communities

16S rRNA sequencing from 16 rhizosphere samples generated a total of 807,695 raw reads. Data denoising and chimera screening was carried out. A total of 10,771 joined read pairs per sample were used for identification. The sequences with >97% similarity were combined by classification into operational taxonomic units (OTUs). The OTUs were assigned to 48, 126, 264, 380, and 1148 taxa at the phylum, class, order, family, and genus levels, respectively.

**Figure 4:**
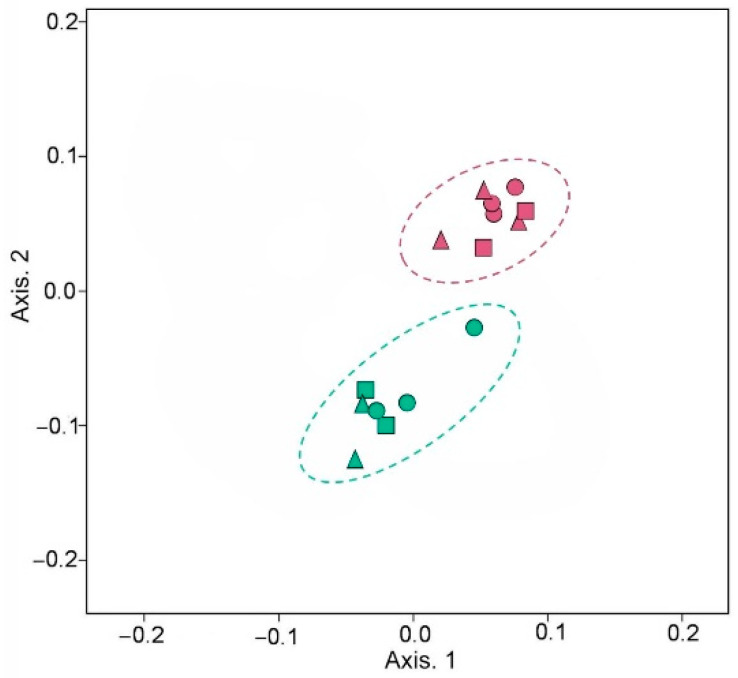
Principal coordinate analysis for the rhizosphere microbial communities of *M. × giganteus*: circles (●), non-inoculated; triangles (▲), *Mycolicibacterium* sp. Pb113; squares (■), *Chitinophaga* sp. Zn19; pink, uncontaminated soil; green, Zn-contaminated soil.

α- and β-Diversity calculations were performed to assess the richness of the microbial communities and characterize the microbial diversity (Table 2 and Figure 4).

The α-diversity was measured by using Chao1, Simpson, and Shannon species richness indices and phylogenetic diversity (Faith’s PD) (Table 2). Shannon’s, Simpson’s, and Faith’s PD indices of bacterial α-diversity indicate that the microbial communities of the rhizosphere of the inoculated plants were more diverse than those for the non-inoculated ones.

The influence of soils on the formation of rhizosphere communities was revealed by comparing the microbiomes of the rhizosphere of different samples. Principal coordinate analysis showed differences in the formation of the taxonomic structure of the miscanthus rhizosphere microbiomes, which was largely affected by the zinc contamination of soil.

#### 3.3.2. Taxonomic Structure of Rhizosphere Microbial Communities

The results of MiSeq sequencing showed that the miscanthus rhizosphere communities included 1148 genera of bacteria belonging to 380 families of 48 phyla.

**Figure 5:**
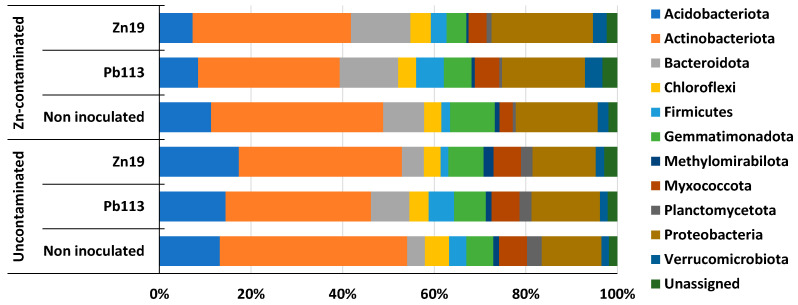
The relative abundances of OTUs associated at the phylum level of the microbial communities in the rhizosphere of *M. × giganteus* non-inoculated and inoculated with heavy-metal-resistant PGPR.

Figure 5 represents the relative abundances of OTUs associated at the phylum level in the rhizosphere of *M. × giganteus* non-inoculated and inoculated with the heavy-metal-resistant PGPR studied. In the rhizosphere communities of *M. × giganteus*, most OTUs were associated with the Actinobacteriota (31–41%), Proteobacteria (13–22%), Acidobacteriota (7–17%), Bacteroidota (4–13%), and Gemmatimonadota (4–10%). The number of OTUs assigned to other phyla was much smaller.

Soil contamination with zinc had a significant effect on the taxonomic profile of the rhizosphere microbiome of both inoculated and non-inoculated *M. × giganteus* plants. Under the influence of zinc in the rhizosphere of plants non-inoculated and inoculated with *Mycolicibacterium* sp. Pb113 or *Chitinophaga* sp. Zn19, the abundance of Proteobacteria increased by 37%, 12%, and 61%, respectively (*p* ≤ 0.0003), and that of Bacteroidota increased by 133%, 52%, and 170%, respectively, (*p* ≤ 0.0003). Yet, Zn contamination decreased the abundance of: Acidobacteriota by 14%, 41%, and 58%, respectively (*p* ≤ 0.0005); Methylomirabilota by 15%, 47%, and 77%, respectively (*p* ≤ 0.004); Myxococcota by 54%, 11%, and 35%, respectively (*p* ≤ 0.002); and Planctomycetota by 78%, 78%, and 56%, respectively (*p* ≤ 0.04). The proportion of the dominant Actinobacteriota type under the influence of zinc decreased insignificantly (by 2–8%). Analysis of changes in the taxonomic profile of the rhizosphere communities under the influence of the inoculant strains revealed a significant (*p* ≤ 0.05) promoting effect of *Mycolicibacterium* sp. Pb113 for the Firmicutes phylum, only compared to non-inoculated plants.

The family-level taxa for which the share in the rhizosphere microbiome of miscanthus was ≥1% are listed in Table S1.

**Figure 6:**
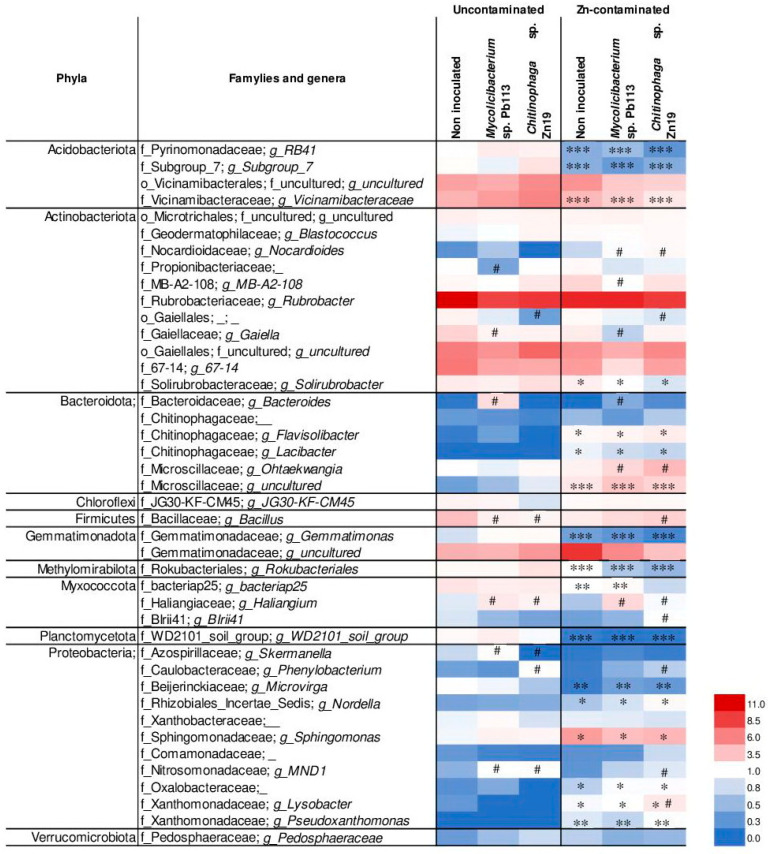
Heat map of the relative abundances of OTUs associated at the genus level of the rhizosphere microbial communities of *M. × giganteus* non-inoculated and inoculated with heavy-metal-resistant PGPR. The differences between uncontaminated and Zn-contaminated rhizospheres are significant at *p* < 0.05 (*), *p* < 0.005 (**), and *p* < 0.001 (***). The differences between non-inoculated and inoculated rhizospheres are significant at *p* < 0.05 (#).

The dominant Actinobacteriota phylum included 31 families, 11 of which were the most abundant (Table S1, Figure 6), making up 82 to 90% of all detected actinobacterial families in the miscanthus rhizosphere. Within the Actinobacteriota, Rubrobacterales was the most abundant order (12%) followed by Gaiellales (10%) and Solirubrobacterales (9%) in the rhizosphere microbiome of *M. ×giganteus*. *Rubrobacter, 67-14* genus, and the genus of uncultured bacteria of the Gaiellales order were the dominant taxa of the Actinobacteria phylum. These taxa prevailed in all treatments, regardless of Zn contamination and microbial inoculation of the rhizosphere soil. A negative correlation was observed between *Solirubrobacter* and metal contamination of soil (*r_s_* = −0.579; *p* < 0.05). The abundance of this genus decreased significantly in Zn-contaminated soil non-inoculated (by 9%) and inoculated with *Mycolicibacterium* sp. Pb113 (by 33%) or *Chitinophaga* sp. Zn19 (by 64%). The effect of the inoculants on the abundance of some taxa of the Actinobacteriota was revealed. *Mycolicibacterium* sp. Pb113 increased the abundance of Nocardioides but significantly (*p* < 0.05) decreased the abundance of Propionibacteriaceae, MB-A2-108, and *Gaiella* both in uncontaminated and Zn-contaminated soil. In contrast, Pb133 increased the abundance of Nocardioides in Zn-contaminated soil. *Chitinophaga* sp. Zn19 significantly (*p* < 0.05) decreased the abundance of an unknown taxon of the Gaiellales order.

There was a downward trend in the abundance of the Mycobacteriaceae family under the influence of both inoculation (including with *Mycolicibacterium* sp. Pb113) and zinc contamination. Moreover, representatives of this taxon were found only in non-inoculated and Pb113-treated uncontaminated soil and were not found at all in Zn-contaminated soil (Table S1).

Seventy-five bacterial families in another dominant phylum, Proteobacteria, were identified. Thirteen of them (Table S1, Figure 6) made the largest contribution to the structure of the miscanthus rhizosphere microbiome. The major class of the phylum was Alphaproteobacteria. Among this class, the dominant position was occupied by the families Sphingomonadaceae (up to 66% of all Alphaproteobacteria families), Beijerinckiaceae (up to 29%), and Xanthobacteraceae (up to 22%). A large proportion also belonged to the Azospirillaceae (up to 20%), Caulobacteraceae (up to 34%), and Rhizobiales Incertae Sedis families. The Nitrosomonadaceae, Comamonadaceae, and Oxalobacteraceae families were predominant members of the Betaproteobacteria class. The most abundant family of the Gammaproteobacteria was Xanthomonadaceae. The rhizosphere proteobacteria of M. × giganteus were sensitive to the presence of zinc. The abundance of the *Sphingomonas*, *Nordella*, and *Lysobacter* genera significantly increased in Zn-contaminated soil (by 4.9, 2.2, and 2.6-fold, respectively). The *Pseudoxanthomonas* genus was absent in uncontaminated rhizosphere soil but present in Zn-contaminated soil.

The close and strong positive correlations between Zn contamination and the abundance of the *Sphingomonas* (*r_s_* = 0.63, *p* < 0.05), *Nordella* (*r_s_* = 0.58, *p* < 0.05), *Lysobacter* (*r_s_* = 0.70, *p* < 0.05), and *Pseudoxanthomonas* (*r_s_* = 0.81, *p* < 0.005) genera were found (Figure 6). In contrast, the abundance of the *Skermanella*, *Phenylobacterium*, and *Microvirga* genera decreased significantly under the influence of metal contamination of soil. A strong negative correlation (*r_s_* = 0.77, *p* < 0.005) between the abundance of the *Microvirga* genus and Zn contamination was observed. Inoculation of plants with *Mycolicibacterium* sp. Pb113 significantly (*p* < 0.05) increased the abundance of the *Skermanella* and MND1 genera in uncontaminated soil, whereas inoculation with *Chitinophaga* sp. Zn19 significantly (*p* < 0.05) increased the relative abundance of the MND1 and *Phenylobacterium* genera both in uncontaminated and in Zn-contaminated soil. A close correlation between inoculation and abundance of the MND1 (*r_s_* = 0.63, *p* < 0.05) and *Phenylobacterium* (*r_s_* = 0.68, *p* < 0.05) genera was found.

The rhizosphere microbiome of *M. × giganteus* was enriched with members of the Acidobacteriota phylum, among which the Vicinamibacteraceae, an uncultured family of the Vicinamibacterales order, Pyrinomonadaceae, and Subgroup_7 families predominated, making up 80% to 91% of all Acidobacteriota families detected in all treatments (Table S1, Figure 6). *Vicinamibacteraceae*, *Subgroup_7*, and *RB41* were the predominant genera of the phylum. Zn contamination of soil clearly reduced the abundance of Acidobacteriota in the microbial communities of the miscanthus rhizosphere. A strong negative correlation was found between Zn contamination and the abundance of the Vicinamibacteraceae, *Subgroup_7*, and *RB41* (*r_s_* = −0.87, *p* < 0.001).

A considerable part of the microbial communities of the miscanthus rhizosphere were members of the Bacteroidota phylum. Chitinophagaceae and Microscillaceae were its most abundant families. This phylum was susceptible to heavy metal contamination, which was manifested as a strong increase in the relative abundance of all major taxa. The close positive correlations between Zn contamination and the abundance of *Flavisolibacter* (*r_s_* = 0.66, *p* < 0.05), *Lacibacter* (*r_s_* = 0.69, *p* < 0.05), and an uncultured genus of the Microscillaceae family (*r_s_* = 0.87, *p* < 0.001) were found. The inoculation of plants with *Mycolicibacterium* sp. Pb113 significantly (*p* < 0.05) increased the abundance of Bacteroides both in uncontaminated and in Zn-contaminated soil. Both inoculants promoted the growth of *Ohtaekwangia* in Zn-contaminated soil.

The Gemmatimonadota phylum, mainly represented by the Gemmatimonadaceae family (78–96% of all families of the phylum), also made a significant contribution to the formation of the miscanthus rhizobiome in all treatments (Figure 6). The abundance of the uncultured genus of the Gemmatimonadaceae reached 3.9–8.7% in the rhizosphere microbial communities. The relative abundance of this taxon doubled in the rhizosphere of non-inoculated plants under the influence of Zn; however, plant inoculation reduced this effect.

The Myxococcota phylum also occupied a prominent position in the miscanthus rhizobiome. Both inoculants were found to increase the relative abundance of its representative *Haliangium* both in uncontaminated and in Zn-contaminated soil.

### 3.4. Effect of Bacterial Inoculants on Zn Content in Plant Biomass

**Figure 7:**
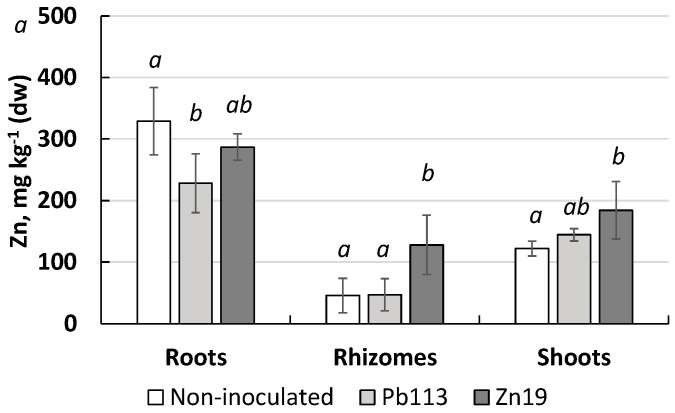
Zn content in biomass of *M. × giganteus* non-inoculated and inoculated with PGPR. 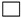
—non-inoculated; 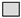
—*Mycolicibacterium* sp. Pb113; 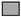
—*Chitinophaga* sp. Zn19. Values represent means, and bars represent confidence interval (n ≥ 3); different letters mean significant difference between treatments at *p* ≤ 0.05.

The results of zinc content analysis in the miscanthus biomass are given in Figure 7.

It was found that the non-inoculated plants accumulated Zn mainly in the roots (329 mg/kg), more than half as much in the leaves (125 mg/kg) and even less in the rhizomes (46 mg/kg). The TF value was 0.37, indicating phytostabilization as the principal mechanism of soil phytoremediation from Zn. The treatment of miscanthus with the metal-resistant PGPR revealed differences in the effect of the inoculants on Zn accumulation in plant biomass. Thus, the strain *Mycolicibacterium* sp. Pb113 decreased Zn content in the roots but had no significant effect on metal content in the rhizomes and leaves, significantly increasing the TF to 0.63. The strain *Chitinophaga* sp. Zn19 increased the metal content in the leaves (by 51%) and rhizomes (by 180%) without affecting metal accumulation in the roots. As a result, the TF increased to 0.64.

## 4. Discussion

The efficacy of *M. × giganteus* in the rehabilitation of soils contaminated by heavy metals is well known (e.g., [5,6,46,47]). However, despite the resistance of this plant to heavy metals, its growth and biomass accumulation may be weakened under conditions of heavy pollution [48,49], which may decrease its remediation potential. Under such conditions, the role of indigenous and/or introduced rhizosphere microorganisms able to be associated with the plant is increased. Being resistant to pollutants and able to promote plant growth, such microorganisms can compensate for the stressful effects of pollutants on plants. On the basis of this, plant-assisted phytoremediation is currently a promising approach to soil remediation in situ, as compared with traditional bio- and phytoremediation [12,26,50]. In this regard, the isolation and examination of promising microbial inoculants for remediating plants is of great importance.

During a microbiological study using traditional cultural methods, we obtained isolates Pb113 and Zn19 from the rhizosphere of *M. × giganteus* grown in soil contaminated with heavy metals [32,34]. The isolates showed plant-growth-promoting properties and were resistant to heavy metals. In this research, the isolates were identified as *Mycolicibacterium* sp. Pb113 and *Chitinophaga* sp. Zn19. The subsequent metagenomic analysis of the rhizospheric microbial communities of *M. × giganteus* confirmed the presence of taxa corresponding to *Mycolicibacterium* sp. Pb113 (d_Bacteria; p_Actinobacteriota; c_Actinobacteria; o_Corynebacteriales; f_Mycobacteriaceae; g_Mycobacterium) and *Chitinophaga* sp. Zn19 (d_Bacteria; p_Bacteroidota; c_Bacteroidia; o_Chitinophagales; f_Chitinophagaceae; g_Chitinophaga) in the root zone of this plant (Table S1).

The presence of PGPR traits in a microbial candidate for association with *M. × giganteus* is important, because in the case of its application, biomass growth in the remediator plant is expected to increase, as is the efficacy of soil cleanup. The strains we had isolated manifested such signs of potential PGPR. On the basis of the obtained data, it can be assumed that these microorganisms would be able to promote the growth of miscanthus owing to nitrogen fixation and to the production of the phytohormone IAA and siderophores. Such properties have been described previously for *Chitinophaga* spp. and *Mycolicibacterium* spp. [51,52,53]. However, the use of such inoculants can be effective only if they survive in polluted soil, which should be due to their resistance to the toxic effect of the pollutant. Therefore, the resistance of the strains to heavy metals in the environment was an important selection criterion. In testing the microorganisms, we found that they were resistant to zinc and lead (Table 1), in agreement with the previously reported data on the resistance of bacteria of the same genera *Chitinophaga* [54] and *Mycolicibacterium* [55,56] to heavy metals. On the basis of their properties, *Mycolicibacterium* sp. Pb113 and *Chitinophaga* sp. Zn19 have been tested as inoculants in a pot trial to promote the growth and remediation potential of *M. × giganteus* grown in Zn-contaminated soil.

The results of the experiment showed that the inoculation of miscanthus with *Mycolicibacterium* sp. Pb113 and *Chitinophaga* sp. Zn19 in clean soil contributed to increased growth, tillering, and biomass accumulation of *M. × giganteus*, and the effect of inoculation with *Mycolicibacterium* sp. Pb113 was greater. The phytotoxicity of the Zn-contaminated soil was manifested as a decrease in plant height, whereas no significant inhibition of *M. × giganteus* biomass accumulation was observed (Figure 2 and Figure 3). Under pollution conditions, inoculation of miscanthus with *Chitinophaga* sp. Zn19 resulted in an increase in tillering but not in plant height, as well as a 50% increase in aboveground biomass accumulation. The effect of inoculation with *Mycolicibacterium* sp. Pb113 was manifested as an increase in the height and tillering of plants, as well as an increase in the accumulation of both aboveground and belowground biomass by 54 and 34%, respectively. The results obtained are comparable to and in some cases surpass the previously reported data on the inoculation of *Miscanthus* spp. with PGPR belonging to various taxa [26,27,28,29,30,31]. A positive effect of the PGPR strain *Pseudomonas koreensis* AGB-1 on the growth of *M. sinensis* in a metal-contaminated mining soil, which was an increase in the plant biomass of 41.6%, was reported by [26]. Fei et al. observed a significant increase in shoot weight in *M. × giganteus* cv. Amuri treated with *Gluconacetobacter diazotrophicus* PAL5T LsdB++, *Gluconacetobacter johannae* UAP-Cf-76, and *Variovorax paradoxus* JM67 by 15–24%, as compared with the untreated controls [28]. Similar positive effects were observed by Schmidt et al. [27], when *M. × giganteus* was treated, before planting, with RhizoPlus^®^, a commercial formulation containing *Bacillus amyloliquefaciens* strain FZB24. According to [29], the treatment of *M. × giganteus* with the PGPR *Bacillus altitudinis* KP-14 resulted in a significant increase in total shoot and root weight by 77.7% and 55.5%, respectively. Recently, Pidlisnyuk et al. also observed a positive effect of three bacterial strains (*Stenotrophomonas maltophilia* KP-13, *B. altitudinis* KP-14, and *Pseudomonas fluorescens* KP-16), both singly and in combination, on leaf biomass productivity in *M. × giganteus* [31]. A positive effect of miscanthus inoculation with *Herbaspirillum frisingense* GSF30T was observed by [30].

In this study, the promotion of miscanthus growth by members of the *Mycolicibacterium* and *Chitinophaga* genera was described for the first time. The ability of mycobacteria to promote the growth of plants was reported previously for *Dendrobium moschatum* [57]; *Brassica napus* [58]; *Triticum* sp., *Zea mays*, *Pisum sativum*, and *Gossypium hirsutum* [59]; *Sorghum bicolor* [60]; a mixture of *Festuca arundinacea*, *F. elata*, and *F. gigantean* [61]; *Trifolium repens* [62]; and *Medicago sativa* [39]. The presence of signs of PGPR, including the synthesis of the phytohormone IAA, the dissolution of phosphates, the synthesis of ACC deaminase, in members of *Chitinophaga* spp. has also been described previously [63,64].

The beneficial effects of microbial inoculation can be driven indirectly through effects on the diversity and composition of the resident plant rhizosphere microbiome [30,65]. Therefore, in our study, we paid special attention to the study of the influence of the pollutant and inoculant strains on the structure of the rhizosphere microbial communities of *M.× giganteus*.

Analysis of the rhizosphere microbiome of *M. ×giganteus* allowed us to identify taxa with potential functions in plant growth promotion and assisted phytoremediation. Potential PGPR taxa included *Sphingomonas* (Proteobacteria, Sphingomonadaceae [66]), *Phenylobacterium* (Proteobacteria, Caulobacteraceae [66,67]), Azospirillaceae family (Proteobacteria [68]), *Bacillus* (Firmicutes, Bacillaceae [27,29,69,70]), *Gemmatimonas* (Gemmatimonadota, Gemmatimonadaceae [70]), and *Haliangium* (Myxococcota Haliangiaceae [71,72,73]). Inoculation of miscanthus has contributed to an increase in the number of some of these taxa, in particular, the *Bacillus*, *Haliangium*, and *Sphingomonas* genera. Thus, the inoculants could promote plant growth in polluted soil not only directly but also indirectly, thus affecting the number of populations interacting with the plant.

Along with the characteristics of plant species, soil environment is responsible for the formation of microbial communities in the plant rhizosphere [74,75]. The results of the study showed that the microbial communities of miscanthus were represented by the dominant Proteobacteria, Acidobacteriota, as well as the Bacteroidota and Gemmatimonadota phyla. The features of the rhizobiomes of miscanthus grown in the soil type studied was the predominance of *Rubrobacter*, *67-14* and uncultivated bacteria of the order Gaiellales (Actinobacteriota), uncultivated bacteria of the Gemmatimonadaceae family (Gemmatimonadota), as well as Vicinamibacteraceae and uncultivated bacteria of the Vicinamibacteraceae family (Acidobacteriota). The high enrichment of the miscanthus rhizobiomes by members of these taxa was maintained regardless of the treatment used (Zn contamination or PGPR inoculation). Of interest, the identified taxa included *Rubrobacter*, whose members differ in their tolerance to extreme environmental conditions such as radiation and elevated temperature [76]. Some authors have noted a correlation between the presence of *Rubrobacter* members in the environment and metal contamination [77,78]. However, in our study, the abundant of this genus was found both in Zn-contaminated and in uncontaminated soil.

Changes in the composition and structure of the soil microbial community of the *M. × giganteus* rhizosphere under the influence of metals has also been reported in a number of works [18,21,22,24,70]. Various changes were noted in the taxonomic profile of the miscanthus rhizobiome, which may be due to the different soil types used in research, the duration of plant cultivation, and other factors. According to the data obtained, soil contamination with Zn had a pronounced influence on the formation of the rhizosphere microbiome of miscanthus (Figure 4). This was manifested mainly as a decrease in the abundance of Acidobacteriota, as well as Methylomirabilota, Myxococcota, and Planctomycetota, and a simultaneous increase in the abundance of Proteobacteria and Bacteroidota. The indicator taxa whose abundance decreased most markedly under the influence of soil contamination with Zn were RB41, Subgroup_7 and Vicinamibacteraceae (Acidobacteriota), Gemmatimonas (Gemmatimonadota), bacteriap25 (Myxococcota), WD2101_soil_group (Planctomycetota), and *Microvirga* (Proteobacteria). Yet, the number of other taxa clearly increased—an uncultured genus of the Microtrichales order (Actinobacteriota), *Nocardioides* (Actinobacteriota), *Flavisolibacter*, *Lacibacter*, *Ohtaekwangia*, and an uncultured genus of the Microscillaceae family (Bacteroidota), *Nordella*, *Lysobacter*, *Pseudoxanthomonas*, and especially *Sphingomonas* (Proteobacteria). Under the influence of the inoculants in polluted soil, the number of *Bacillus* (Firmicutes) increased. Many of the identified taxa can take part in the remediation of soil from Zn. According to the literature, such properties are possessed by members of *Bacillus* [79], *Sphingomonas* [70,80], and *Pseudoxanthomonas* [81].

Interestingly, the taxa corresponding to the inoculants used reacted differently to soil contamination: the abundance of the Mycobacteriaceae family decreased, and the abundance of the Chitinophagaceae family increased significantly in response to Zn treatment of soil (Table S1, Figure 6). In this connection, we assume that the effect of inoculants on the growth and phytoremediation efficiency of miscanthus could be direct (probably for *Chitinophaga* sp. Zn19) and indirect (mainly for *Mycolicibacterium* sp. Pb113) through the impact on other rhizospheric microorganisms.

Inoculation of *M. ×giganteus* with the PGPR strains used improved the phytoremediation ability of the plant. All the rhizobacteria tested increased Zn uptake by miscanthus, enhancing the bioaccumulation and translocation of the metal to the aboveground organs, as evidenced by a rising the TF more than 70%. *Chitinophaga* sp. strain Zn19 was characterized by the highest efficiency, increasing the translocation of the metal into plant leaves by 180%, as compared with the non-inoculated control. Thus, the use of bacterization of plants allowed us to increase the removal of Zn from roots to shoots. We speculate that under conditions of longer cultivation of miscanthus, this technique will contribute to improving the cleanup of soil contaminated with Zn. The bacteria used in this study, after being tested for virulence and pathogenicity, can be recommended for use to improve the efficiency of the phytoremediation of Zn-contaminated soil.

## 5. Conclusions

A positive effect of inoculation with two heavy-metal-resistant PGPR, *Chitinophaga* sp. Zn19 and *Mycolicibacterium* sp. Pb113, on the growth, rhizosphere microbiome, and remediation ability of *Miscanthus × giganteus* was revealed. *Mycolicibacterium* sp. Pb113 significantly promoted the accumulation of the aboveground and belowground biomass of miscanthus in uncontaminated as well as in Zn-contaminated soil. *Chitinophaga* sp. Zn19 enhanced the accumulation of aboveground biomass in Zn-contaminated soil. *Mycolicibacterium* sp. Pb113 reduced Zn accumulation in roots and had no effect on metal accumulation in rhizomes and in leaves. *Chitinophaga* sp. Zn19 increased the Zn content in leaves and rhizomes. The inoculation of plants with heavy-metal-resistant bacteria increased the translocation of Zn from roots to shoots. *Chitinophaga* sp. Zn19 showed the highest translocation of Zn into plant leaves, as compared with the non-inoculated control. Evaluation of the impact of Zn contamination and inoculation of plants with the bacteria studied on the structure and diversity of the rhizosphere microbial communities showed differences in the formation of the miscanthus rhizosphere microbiomes, which was affected by Zn rather than by the inoculants. Zn contamination of soil reduced the abundance of Acidobacteriota, Methylomirabilota, Myxococcota, and Planctomycetota and simultaneously increased the abundance of Proteobacteria and Bacteroidota. Bacterial taxa that are potentially involved in the promotion of plant growth and in assisted phytoremediation were identified. The effect of miscanthus inoculation with *Chitinophaga* spp. and *Mycolicibacterium* spp. was shown for the first time. On the basis of the obtained data, the bacterial strains studied may be recommended for use to improve the efficacy of *M. × giganteus* remediation of Zn-contaminated soil.

## Figures and Tables

**Table 1:** Heavy metal resistance and PGPR traits of the bacteria studied.

Properties	Pb113	Zn19
PGPR traits:		
nitrogen fixation	+	+
phosphorous mobilization	−	−
siderophore synthesis	+	+
IAA production	+	+
Metal resistance (MIC, mmol/L)		
Zn	3.0	2.5
Pb	≥5.0	2.0

Notes: “+”, found; “−”, not found.

**Table 2:** The α-diversity indices for the rhizospheric microbial communities of *M. × giganteus*.

Soil	Inoculation	Observed Features	Chao1	Shannon Index	Simpson Index	Faith PD
Uncontaminated	Non-inoculated	294 ± 64	293.60 ± 64.31	7.5813 ± 0.2880	0.9928 ± 0.0013	29.96 ± 7.95
	Pb113	378 ± 16	379.38 ± 16.09	8.0089 ± 0.0316	0.9949 ± 0.0000	32.52 ± 2.96
	Zn19	298 ± 5	298.10 ± 4.10	7.6623 ± 0.0471	0.9935 ± 0.0002	28.10 ± 2.20
Zn-contaminated	Non-inoculated	294 ± 16	295.17 ±17.21	7.5218 ± 0.0345	0.9922 ± 0.0003	26.78 ± 5.88
	Pb113	346 ± 14	347.55 ± 14.42	7.7014 ± 0.0351	0.9930 ± 0.0018	36.66 ± 5.39
	Zn19	414 ± 6	414.56 ± 6.29	7.9681 ± 0.0554	0.9942 ± 0.0007	30.41 ± 3.01

## Data Availability

The Pb113 and Zn19 16S rRNA gene sequences as well as the raw sequence reads can be accessed via NCBI archives with IDs OQ680140, OQ680520, and PRJNA973256, respectively.

## Supplementary Materials

The following supporting information can be downloaded at: https://www.mdpi.com/article/10.3390/microorganisms11061516/s1, Table S1: Taxonomic structure of the rhizosphere microbial communities of non-inoculated and PGPR-inoculated *Miscanthus × giganteus*, cultivated in uncontaminated and Zn-contaminated soil, %.
