# Supplementary material for: Effect of Heavy-Metal-Resistant PGPR Inoculants on Growth, Rhizosphere Microbiome and Remediation Potential of Miscanthus × giganteus in Zinc-Contaminated Soil
Source: Microorganisms. 2023 Jun 7;11(6):1516. doi: 10.3390/microorganisms11061516 (PMC10304086; doi:10.3390/microorganisms11061516)
Supplement: Supplementary file 1 [file microorganisms-11-01516-s001.zip › microorganisms-2366131-supplementary.pdf]

Table S1. Taxonomic structure of rhizosphere microbial communities of non-inoculated and PGPR inoculated *Miscanthus × giganteus*, cultivated in uncontaminated and Zn-contaminated soil, %

OTUs associated at the family level	Uncontaminated soil			Zn-contaminated soil		
	Non-inoculated	Mycolicibacterium sp. Pb113	Chitinophaga sp. Zn19	Non-inoculated	Mycolicibacterium sp. Pb113	Chitinophaga sp. Zn19
d__Bacteria;p__Acidobacteriota;c__Blastocatellia;o__Pyrinomonadales;f__Pyrinomonadaceae	1.15 ± 0.03	1.85 ± 0.11	1.62 ± 0.51	0.43 ± 0.06	0.55 ± 0.11	0.24 ± 0.10
d__Bacteria;p__Acidobacteriota;c__Holophagae;o__Subgroup_7;f__Subgroup_7	1.17 ±0.07	0.95 ± 0.37	2.21 ± 0.30	0.45 ± 0.29	0.29 ± 0.12	0.42 ± 0.20
d__Bacteria;p__Acidobacteriota;c__Vicinamibacteria;o__Vicinamibacterales;f__uncultured	4.71 ± 0.57	4.25 ± 1.38	5.51 ± 0.89	5.23 ± 1.20	3.16 ± 1.05	2.89 ± 0.93
d__Bacteria;p__Acidobacteriota;c__Vicinamibacteria;o__Vicinamibacterales;f__Vicinamibacteraceae	4.21 ± 0.02	5.42 ± 0.72	5.90 ± 0.27	3.92 ± 0.25	3.13 ± 0.34	2.53 ± 0.52
d__Bacteria;p__Actinobacteriota;c__Acidimicrobiia;o__Microtrichales;f__uncultured	1.71 ± 0.05	1.39 ±0.10	1.54 ± 0.48	1.44 ± 0.05	1.55 ± 0.12	1.30 ± 0.23
d__Bacteria;p__Actinobacteriota;c__Actinobacteria;o__Corynebacteriales;f__Mycobacteriaceae	0.14 ± 0.04	0.06 ± 0.02	0.00	0.00	0.00	0.00
d__Bacteria;p__Actinobacteriota;c__Actinobacteria;o__Frankiales;f__Geodermatophilaceae	1.21 ± 0.72	1.04 ± 0.23	1.47 ± 0.62	1.25 ± 0.39	1.20 ± 0.13	1.45 ± 0.58
d__Bacteria;p__Actinobacteriota;c__Actinobacteria;o__Micromonosporales;f__Micromonosporaceae	0.67 ± 0.33	1.70 ± 0.26	0.00	0.62 ± 0.21	1.08 ± 0.32	0.00
d__Bacteria;p__Actinobacteriota;c__Actinobacteria;o__Propionibacteriales;f__Nocardioidaceae	0.23 ± 0.03	0.87 ± 0.28	0.18 ± 0.06	1.09 ± 0.39	1.39 ± 0.41	2.02 ± 0.41
d__Bacteria;p__Actinobacteriota;c__Actinobacteria;o__Propionibacteriales;f__Propionibacteriaceae	1.37 ± 0.37	0.36 ± 0.11	1.59 ± 0.83	1.73 ± 0.09	1.24 ± 0.13	1.35 ± 0.01
d__Bacteria;p__Actinobacteriota;c__MB-A2-108;o__MB-A2-108;f__MB-A2-108	1.36 ± 0.11	1.07 ± 0.22	2.02 ± 0.87	2.70 ± 0.69	1.10 ± 0.14	2.20 ± 0.07
d__Bacteria;p__Actinobacteriota;c__Rubrobacteria;o__Rubrobacterales;f__Rubrobacteriaceae	10.71 ± 1.54	8.14 ± 0.70	8.63 ± 2.18	9.17 ± 2.43	9.17 ± 1.75	8.44 ± 1.09
d__Bacteria;p__Actinobacteriota;c__Thermoleophilia;o__Gaiellales;__	1.57 ± 0.51	0.91 ± 0.28	0.33 ± 0.12	1.27 ± 0.76	0.96 ± 0.32	0.86 ± 0.02
d__Bacteria;p__Actinobacteriota;c__Thermoleophilia;o__Gaiellales;f__Gaiellaceae	2.79 ± 0.42	1.54 ± 0.31	1.56 ± 0.38	1.83 ± 0.29	0.71 ± 0.15	1.52 ± 0.51
d__Bacteria;p__Actinobacteriota;c__Thermoleophilia;o__Gaiellales;f__uncultured	6.12 ± 1.10	5.25 ± 0.92	6.91 ± 3.54	5.32 ± 1.07	4.25 ± 1.10	5.61 ± 0.10
d__Bacteria;p__Actinobacteriota;c__Thermoleophilia;o__Solirubrobacterales;f__67-14	6.26 ± 0.11	4.46 ± 1.88	4.37 ± 1.64	4.85 ± 0.39	2.38 ± 1.00	4.71 ± 0.65
d__Bacteria;p__Actinobacteriota;c__Thermoleophilia;o__Solirubrobacterales;f__Solirubrobacteraceae	2.82 ± 0.17	2.27 ± 0.35	2.72 ± 0.10	2.07 ± 0.16	1.34 ± 0.32	1.31 ± 0.11
d__Bacteria;p__Bacteroidota;c__Bacteroidia;o__Chitinophagales;f__Chitinophagaceae	1.35 ± 0.18	1.71 ± 0.18	1.98 ± 0.18	4.34 ± 2.03	4.05 ± 2.71	4.46 ± 0.69
d__Bacteria;p__Bacteroidota;c__Bacteroidia;o__Cytophagales;f__Microscillaceae	1.62 ± 0.63	1.62 ± 0.08	2.22 ± 1.10	3.70 ± 0.03	6.22 ± 1.02	7.35 ± 1.86
d__Bacteria;p__Chloroflexi;c__Chloroflexia;o__Thermomicrobiales;f__JG30-KF-CM45	1.58 ±0.61	1.46 ±0.08	0.84 ±0.04	1.26 ±0.21	1.39 ±0.19	1.31 ±0.61
d__Bacteria;p__Chloroflexi;c__Gitt-GS-136;o__Gitt-GS-136;f__Gitt-GS-136	0.57 ±0.41	0.70 ±0.27	0.81 ±0.06	1.00 ±0.25	0.77 ±0.16	0.62 ±0.09
d__Bacteria;p__Firmicutes;c__Bacilli;o__Bacillales;f__Bacillaceae	3.53 ±1.56	1.68 ±0.09	1.53 ±0.42	1.88 ±0.23	2.54 ±0.21	2.86 ±1.68
d__Bacteria;p__Gemmatimonadota;c__Gemmatimonadetes;o__Gemmatimonadales;f__Gemmatimonadaceae	5.18 ±0.02	6.13 ±1.46	6.75 ±3.26	9.04 ±0.50	5.88 ±0.34	3.94 ±3.11
d__Bacteria;p__Methylomirabilota;c__Methylomirabilia;o__Rokubacteriales;f__Rokubacteriales	1.24 ±0.04	1.29 ±0.24	2.18 ±0.51	1.06 ±0.06	0.64 ±0.08	0.50 ±0.12
d__Bacteria;p__Myxococcota;c__bacteriap25;o__bacteriap25;f__bacteriap25	2.18 ±0.31	1.75 ±0.22	1.82 ±0.16	1.07 ±0.93	1.24 ±0.16	0.77 ±0.23
d__Bacteria;p__Myxococcota;c__Myxococcia;o__Myxococcales;f__Myxococcaceae	1.08 ±0.82	0.51 ±0.01	1.00 ±0.52	0.48 ±0.25	0.23 ±0.03	0.11 ±0.05
d__Bacteria;p__Myxococcota;c__Polyangia;o__Haliangiales;f__Haliangiaceae	0.89 ±0.42	1.99 ±1.69	1.56 ±1.50	0.62 ±0.41	2.57 ±0.42	1.02 ±0.20
d__Bacteria;p__Myxococcota;c__Polyangia;o__Polyangiales;f__BIrii41	0.85 ±0.43	0.52 ±0.03	0.35 ±0.31	0.34 ±0.17	0.48 ±0.01	1.04 ±0.85
d__Bacteria;p__Nitrospirota;c__Nitrospiria;o__Nitrospirales;f__Nitrospiraceae	0.64 ±0.10	0.54 ±0.13	0.90 ±0.61	0.74 ±0.19	0.31 ±0.01	0.16 ±0.14
d__Bacteria;p__Planctomycetota;c__Phycisphaerae;o__Tepidisphaerales;f__WD2101_soil_group	1.34 ±0.44	1.75 ±0.44	1.02 ±0.22	0.09 ±0.13	0.04 ±0.01	0.06 ±0.05
d__Bacteria;p__Planctomycetota;c__Planctomycetes;o__Gemmatales;f__Gemmataceae	0.95 ±0.22	0.34 ±0.13	0.33 ±0.46	0.02 ±0.03	0.17 ±0.00	0.19 ±0.07
d__Bacteria;p__Planctomycetota;c__Planctomycetes;o__Pirellulales;f__Pirellulaceae	0.57 ±0.44	0.23 ±0.07	0.36 ±0.19	0.44 ±0.02	0.28 ±0.01	0.32 ±0.17
d__Bacteria;p__Proteobacteria;c__Alphaproteobacteria;o__Azospirillales;f__Azospirillaceae	0.77 ±0.38	1.21 ±1.00	0.00 ±0.00	0.06 ±0.09	0.17 ±0.02	0.09 ±0.13
d__Bacteria;p__Proteobacteria;c__Alphaproteobacteria;o__Caulobacterales;f__Caulobacteraceae	0.37 ±0.44	0.24 ±0.34	1.19 ±0.10	0.17 ±0.11	0.55 ±0.02	0.91 ±0.33
d__Bacteria;p__Proteobacteria;c__Alphaproteobacteria;o__Rhizobiales;f__Beijerinckiaceae	1.59 ±1.20	1.53 ±0.69	0.80 ±0.71	0.20 ±0.28	0.61 ±0.03	0.66 ±0.25
d__Bacteria;p__Proteobacteria;c__Alphaproteobacteria;o__Rhizobiales;f__Rhizobiales_Incertae_Sedis	0.42 ±0.03	0.40 ±0.03	0.42 ±0.16	0.72 ±0.03	0.83 ±0.05	1.29 ±0.36
d__Bacteria;p__Proteobacteria;c__Alphaproteobacteria;o__Rhizobiales;f__Xanthobacteraceae	1.25 ±0.59	1.44 ±0.64	0.83 ±0.03	1.07 ±0.06	0.90 ±0.04	1.24 ±0.33
d__Bacteria;p__Proteobacteria;c__Alphaproteobacteria;o__Sphingomonadales;f__Sphingomonadaceae	1.42 ±1.12	2.22 ±0.27	1.63 ±0.49	5.67 ±1.01	4.79 ±0.56	5.02 ±0.01
d__Bacteria;p__Proteobacteria;c__Gammaproteobacteria;o__Burkholderiales;f__Comamonadaceae	0.55 ±0.49	0.60 ±0.28	0.67 ±0.07	1.27 ±0.49	0.25 ±0.03	1.11 ±0.94
d__Bacteria;p__Proteobacteria;c__Gammaproteobacteria;o__Burkholderiales;f__Nitrosomonadaceae	1.02 ±0.02	1.59 ±0.27	2.09 ±0.89	0.44 ±0.04	0.77 ±0.09	0.96 ±0.26
d__Bacteria;p__Proteobacteria;c__Gammaproteobacteria;o__Burkholderiales;f__Oxalobacteraceae	0.41 ±0.03	0.41 ±0.05	0.00 ±0.00	1.01 ±0.30	1.42 ±0.11	1.31 ±0.80
d__Bacteria;p__Proteobacteria;c__Gammaproteobacteria;o__Gammaproteobacteria_Incertae_Sedis;f__Unknown_Family	0.59 ±0.43	0.34 ±0.48	0.69 ±0.12	0.34 ±0.38	0.43 ±0.05	0.68 ±0.58
d__Bacteria;p__Proteobacteria;c__Gammaproteobacteria;o__Steroidobacterales;f__Steroidobacteraceae	0.83 ±0.06	0.80 ±0.25	0.98 ±0.41	0.12 ±0.17	0.18 ±0.03	0.21 ±0.07
d__Bacteria;p__Proteobacteria;c__Gammaproteobacteria;o__Xanthomonadales;f__Xanthomonadaceae	0.28 ±0.20	0.31 ±0.03	0.14 ±0.20	2.04 ±0.30	1.81 ±0.29	3.68 ±2.35
d__Bacteria;p__Verrucomicrobiota;c__Verrucomicrobiae;o__Chthoniobacterales;f__Xiphinematobacteraceae	0.00 ±0.00	0.00 ±0.00	0.00 ±0.00	0.66 ±0.93	0.51 ±0.08	0.73 ±0.04
d__Bacteria;p__Verrucomicrobiota;c__Verrucomicrobiae;o__Opitutales;f__Opitutaceae	0.55 ±0.09	0.49 ±0.06	0.57 ±0.37	0.47 ±0.48	1.26 ±0.16	0.87 ±0.30
d__Bacteria;p__Verrucomicrobiota;c__Verrucomicrobiae;o__Pedosphaerales;f__Pedosphaeraceae	0.30 ±0.43	0.75 ±0.28	1.05 ±0.46	0.82 ±1.00	0.74 ±0.09	0.64 ±0.14
